# Patient external dose rate after ^177^Lu-DOTATATE therapy: factors affecting its decrease and predictive value

**DOI:** 10.7150/ijms.58680

**Published:** 2021-05-17

**Authors:** Jules Zhang-Yin, Nadine Guilabert, Thierry Kiffel, Françoise Montravers, Phillip Calais, Jean Lumbroso, Jean-Noël Talbot

**Affiliations:** 1 Nuclear Medicine, GH Tenon-St Antoine and Sorbonne Université, Paris, France.; 2 Nuclear Medicine and Radiation Safety departments, Gustave Roussy Cancer Campus, Villejuif, France.; 3 Clinical Physics, Royal Perth Hospital, Perth, Australia.

**Keywords:** ^177^Lu-DOTA-octreotate, neuroendocrine tumors, radiation exposure, patient discharge, two-year survival rate

## Abstract

**Rationale:** Peptide receptor radionuclide therapy (PRRT) with ^177^Lu-DOTATATE (oxodotreotide) results in external radiation exposure from the patient. In the PREELU observational prospective study, we determined the equivalent dose rate at 1 m of the patient (EDR-1m) for a period following PRRT. The main objective was to predict which patients could be discharged from the hospital at approximately 3 h after the administration of ^177^Lu-DOTATATE, i.e. at the end of the infusion of amino-acids according to our PRRT protocol. As presenting no undue risk of radiation exposure for the public, those patients could be treated as outpatients or day patients, rather than inpatients.

**Methods:** We sequentially measured EDR-1m facing the sternum and then the pelvis during 50 PRRT in 24 patients with metastatic neuroendocrine tumours, each 30 minutes after ending administration of Lutathera, over at least 180 minutes.

**Results:** 180 minutes after the administration of ca. 7400 MBq of Lutathera, EDR-1m was <40 µSv/h in all cases, and <25 µSv/h in 32 cases (64%). After an overnight hospital stay, EDR-1m was <25 µSv/h in all cases. The EDR-1m value measured facing the sternum was the greatest in about one-fourth of paired measurements. In patients whose creatinine clearance was >96 mL/min/1.73m^2^, the EDR-1m was most likely (predictive value=90%) to drop below 25 µSv/h within 180 minutes after the administration of Lutathera. In 16 patients who benefited from several PRRT cycles, the creatinine clearance did not decrease significantly from one cycle to the next, probably due to the kidney protection by the amino-acid infusion. The patients whose EDR-1m dropped below 25 µSv/h at 180 minutes during their first PRRT cycle were unlikely (predictive value= 88%) to decease during the following two years.

**Conclusion:** All patients could have been discharged 3 h after administration according to the criterion EDR-1m <40 µSv/h. Using EDR-1m <25 µSv/h as criterion, an extended hospital stay beyond 3 h would have been necessary in around one-third of the PRRT treatments and could be anticipated based on creatinine clearance ≤96 mL/min/1.73m^2^. EDR-1m <25 µSv/h at 180 min during the first PRRT yielded a strong predictive value on the patient's survival at two years, a finding that should be confirmed in future studies.

## Introduction

^177^Lu-DOTATATE (oxodotreotide) is currently available as a ready-to-use commercial preparation, Lutathera (Advanced Accelerator Applications, Saint-Genis-Pouilly, France), indicated for the treatment of unresectable or metastatic, progressive, well-differentiated (Grades I and II), somatostatin receptor-positive gastrointestinal neuroendocrine tumours (GEP‑NETs) in adults.

In contrast with non-radioactive somatostatin analogues, cytotoxic agents or other targeted therapies, Lutathera is a radiopharmaceutical medicine which contains a radionuclide, ^177^Lu, suited for internal radiotherapy of GEP-NET lesions. ^177^Lu decays by β^-^ emission to stable Hafnium (^177^Hf) with the most abundant β^-^ (79.3%) having a maximum energy of 497 keV. The average beta energy is approximately 130 keV. Gamma photons are also emitted with an energy of 113 keV (6.2%) and 208 keV (11%). Special precautions are therefore necessary while and after administering Lutathera to a patient, to limit the contamination of the environment from excreted ^177^Lu and also the radiation exposure of the personnel and members of the public due to the gamma photons emission.

Lutathera must thus be administered in a hospital with nuclear medicine facilities and only by persons authorised to handle radiopharmaceuticals. Peptide receptor radionuclide therapy (PRRT) with Lutathera may be implemented either on an inpatient or an outpatient basis. Whereas in Australia 1,2 or Poland 3 ^177^Lu-DOTATATE may be administered on an outpatient basis, in most member states of the European Union, PRRT requires confinement of the patient in a specialized radiation ward for 1-3 days at each administration.

There are several problems raised by such inpatient procedures. These include the higher cost of inpatient as compared to outpatient or day-patient treatment, and limited resource availability such as the number of rooms in radiation wards. Together, these may result in restricting the practice of ^177^Lu PRRT of NETs to a small number of specialized centres and jeopardize the actual role of PRRT in the management of patients with unresectable or metastatic GEP-NET.

Together with the clinical status of the patient after the radiopharmaceutical injection, the radiation exposure to the careers, family and friends of the patient is a determinant factor to decide on the length of the hospital stay of a patient referred for PRRT.

The upper limit of the external equivalent dose rate (EDR) of exposure from a patient who was administered a radiopharmaceutical medicine is derived from the new European Commission Basic Safety Standards Ionising Radiations Regulations 2017 (IRR17) 4, based on recommendations from the International Commission on Radiological Protection (ICRP). They specify that the effective dose limit to a member of public should not exceed 1 mSv per year 5,6, and to ensure this, PRRT patients are kept overnight following therapy. It is common practice to discharge a patient from the hospital when the EDR at 1 m (EDR-1m) from the patient is less than 40 μSv/h 7. However, a lower threshold of 25 μSv/h for EDR-1m is also recommended in some countries 2.

Thus, the hypothesis of a non-systematic overnight stay of patients receiving Lutathera whose EDR-1m is less than the lower threshold must be raised. In this option, it would be important to identify patients for whom a reduced duration of the hospitalization should be scheduled.

The primary objective of this PREELU study was to determine, in a cohort of patients referred to our nuclear medicine departments for Lutathera PRRT, the durations of hospital stay required to obtain EDR-1m less than 40 µSv/h and 25 µSv/h, respectively. In this aim, multiple measurements of EDR-1m were performed during the hours following Lutathera administration. Four secondary study objectives aimed to evaluate factors affecting the EDR-1m decrease, how it may be predicted and its potential predictive value on patient's outcome.

## Materials and methods

We conducted an observational, analytical, prospective study in the two nuclear medicine departments at the Groupe Hospitalier Tenon-St Antoine and the Institut Gustave Roussy (IGR). This PREELU study was approved by the French Medicines Agency (ANSM) on the 19/5/2016, N° 2016-A00897-44.

### Patients

Monitoring of EDR-1m is mandatory after administration of a radiophamaceutical for internal radiotherapy. Thus, performing systematic measurements of EDR-1m at regular time intervals after the end of the PRRT procedure was proposed to all consecutive patients who were treated by Lutathera in the two centres. No patient declined to participate in the trial and no data was discarded.

We anticipated the collection of data from 25 patients, each undergoing an average of two PRRT cycles during the study. In accordance with the current French regulations, administration of the PRRT was followed by an overnight stay of the patient in the nuclear medicine department.

Prior to therapy, patient data was collected, including gender, body mass and mass index (BMI), location of the initial GEP-NET, grade, age at PRRT, as well as clearance of creatinine and imaging workup prior to PRRT. The metastatic spread of the GEP-NET was determined in four regions (liver, lymph nodes, skeleton and peritoneum) using CT and/or MRI and/or FDOPA PET/CT, a sensitive and accurate modality for midgut GEP-NET 8,9, and somatostatin receptor imaging 10 in all cases. The latter is a prerequisite for Lutathera PRRT, to demonstrate overexpression of the somatostatin receptor by well-differentiated GEP-NET lesions. It consisted of ^68^Ga-DOTATOC (edotreotide) PET/CT for the patients from Hôpital St Antoine or of ^111^In-pentetreotide (Octreoscan) scintigraphy for those from IGR.

To reflect the extent of the GEP-NET spread prior to PRRT, we counted the number of metastatic organs (TMO 1 to 4) from the imaging workup. To reflect the overexpression of somatostatin receptor by the GEP-NET lesions, we determined the Krenning score (KS) 11-13 which can be applied to both somatostatin receptor PET and scintigraphy (planar and/or SPECT). It consists of the visual evaluation of most intense abnormal focus and assigning a score: 0, no abnormal focus; 1, very low uptake; 2, uptake less than or equal to that of the liver; 3, uptake greater than the liver; and 4, uptake greater than that of the spleen. Two readers evaluated the KS independently, with a consensus reading in case of a discrepancy. When ^68^Ga-DOTATOC PET/CT had been performed, the maximum value of the maximal standard uptake value (max SUVmax) of the lesions was determined and the total functional tumour volume (TFTV) before the 1^st^ PRRT cycle was determined using 41% isocontour level volumes of interest as described by Velikian et al. 14.

One of the secondary objectives was to determine whether the pattern of the ECR-1m after administration of Lutathera has a predictive value on a patient's outcome. Therefore, follow-up data were collected during at least 24 months after the first PRRT cycle of each patient, to evaluate the patients' overall survival (OS) rate.

### Lutathera PRRT Protocol

Patients were hospitalized in an internal radiation therapy room of the nuclear medicine departments, one day before treatment. To avoid any external contamination of the patient that could be transferred to care givers or members of the public and contribute to their overall radiation exposure, the usual radiation protection precautions were applied (a system for storing excreta and use of disposable material, including disposable pyjamas). A blood test was performed, in particular to determine the creatinine clearance of the patient using the Cockroft's formula 15, and a premedication including oral dexamethasone and aprepitant was administrated as anti-emetic.

On the treatment day, amino-acid solution was infused intravenously over 4 h, to reduce renal tubular reabsorption of Lutathera and thus radiation exposure of the kidneys 16. The infusion of ca. 7400 MBq of^ 177^Lu-DOTATATE (delivered ready-to-use as Lutathera) was started 30 minutes after the start of the amino-acid infusion. The Lutathera infusion was delivered over 30 minutes using a shielded syringe driver, or an automated infusion device (Perfucis), in order to reduce radiation exposure to the administering technologist 17.

Patients were discharged on the following day, after being informed about radiation safety measures and instructions.

### EDR-1m measurements

At Hôpital St Antoine, the EDR-1m measurements were performed using a Target Identifinder radiometer (N/9V ARIES), which is a portable 0.01 to 1 Sv/h dose-rate meter and 1024 channel gamma spectrometer with a NaI (Tl) scintillation detector. At Institut Gustave Roussy, a similar gamma radiation dosimeter, the APVL AT-1123, was used, which has a plastic scintillator. A cross-calibration between these two devices for ^177^Lu detection was performed prior to measurements. The gamma detection was performed using a large energy window that includes the two main ^177^Lu gamma rays. During the measurements, the detector axis was perpendicular to the patient surface, avoiding any different angle. The patients were asked to empty their bladder prior to the measurements 18.

At each time point, the dose-rate measurements consisted of a pair of values, facing the sternum of the patient (st-EDR-1m) and then facing the pelvis (pel-EDR-1m). The highest of the paired values at a time point was called maxEDR-1m.

The first measurement was planned just after the end of Lutathera administration. Subsequent measurements were scheduled every ca. 30 min over 180 min. Additional measurements were performed as appropriate, in particular when the EDR-1m at 180 min was still greater than 25 μSv/h. One final measurement was planned on the following day (D1) prior to the patient's departure.

Since some variations from this time schedule were expected in practice, the actual measurement time points were recorded.

### Data analysis and statistics

The main qualitative variable reflecting the decrease of the radiation exposure rate from the patient was whether or not the maxEDR-1m fell below 25 µSv/h within the 180 min following the end of Lutathera administration. According to this criterion, the series of PRRT cycles were sorted into inf-25@180 if matched and sup-25@180 if unmatched. The quantitative variables reflecting the external radiation exposure were: the time points when maxEDR-1m dropped below 40 µSv/h and 25 µSv/h respectively, the maxEDR-1m value at 180 min, and the maxEDR-1m value at D1. As the exact values of 40 µSv/h and 25 µSv/h were not always observed and due to slight differences in time of measurements, linear interpolation was used to estimate the value for the above-mentioned quantitative variables.

The statistical analysis was performed using the software “MedCalc” (Ostende, Belgium) with a significance level of p<0.05. Receiver operating characteristic (ROC) curve analysis was performed for the comparison of quantitative criteria which could predict the rapidity of the drop of EDR-1m after Lutathera administration, Chi^2^ tests for comparing qualitative variables, Wilcoxon's test for comparing paired quantitative data, Pearson's correlation coefficient to search for a relation between quantitative variables and Kaplan-Meier method for the survival analysis.

## Results

The study started in May 2016 and the objective of analysing 50 PRRT cycles was reached in September 2018, by including 24 patients with a variable number of PRRT cycles.

### Population characteristics

Of the 24 patients, 10 male and 14 female, 16 were treated at Institut Gustave Roussy and 8 at Hôpital St Antoine.

Their mean age at their first PRRT cycle was 59 (SD=10.3, median=59.5, range 33-73). Their mean body mass was 68.2 kg (SD=14.3, median=66, range=47-103) and mean BMI was 22.5 kg/m^2^ (SD=2.4, median=22.6, range=18.9-27) (Table 1).

NETs originated from the midgut in 13 cases, from the foregut in 10 cases (including 7 panNET, 1 duodenal, 1 gastric and 1 bronchial NET), and from the ovary in 1 case; it was graded I in 11 patients, II in 11 patients and III in 2 patients.

The patients' NETs metastasized most frequently to the liver (23/24), followed by lymph nodes (21/24), peritoneum (20/24) and skeleton (17/24). The mean of the total number of metastatic organs (TMO) was 3.4 (median=4, range 1-4) and the mean KS was 2.5 (median=2.5, range=1-4).

In this series were included measurements performed during one single PRRT cycle in 8 patients, two PRRT cycles in 9 patients, three PRRT cycles in 4 patients and four PRRT cycles in 3 patients. The median time interval between two successive courses of one patient was 56 days (range=49-421). Mean creatinine clearance, determined prior to each PRRT course, was 97.7 mL/min/1.73m^2^ (SD=18.9, median=99, range=45-134).

### EDR-1m measurements

The mean injected activity of Lutathera for the 50 PRRT was 7448 MBq (median=7400, range=7208-7743) (Table 2).

After the end of the Lutathera infusion, a total of 385 paired measurements were performed. All patients had at least 7 paired measurements of st-EDR-1m and pel-EDR-1m (mean=7.7, median=7, range=6-13), except one patient for whom 6 paired measurements only were performed because of a poor clinical status, both EDR-1m values being <25 µSv/h at 126 min. The greatest of the two paired values at one time point, noted maxEDR-1m, was considered, in order to maximize the evaluation of the external radiation exposure.

The mean time period to achieve maxEDR-1m <40 μSv/h was 24.2 min (SD=37, median=10, range=0-180). All patients during all therapy administrations had maxEDR-1m <40 μSv/h at 180 min.

MaxEDR-1m <25 μSv/h was achieved within 180 min in 32 treatments (64%), constituting the inf-25@180 group. In 7 treatments, maxEDR-1m fell below <25 μSv/h after 180 min. In those 39 administrations, the mean time to observe maxEDR-1m <25 μSv/h was 141 min (SD=43, median=146, range=43-239). For the remaining 11 treatments, the maxEDR-1m was still ≥25 μSv/h at the end of the measuring period (mean time=216 min, median=210, range=183-250): its mean value was then 30 μSv/h (SD=3.8, median=28, range=25-38). Altogether, those 18 treatments (38%) constituted the sup-25@180 group which included 9 of the 30 treatments for midgut NET (30%) vs. 9 of the 19 treatments (47%) for foregut NET (Chi^2^=2.1, p=0.35).

The mean of maxEDR-1m at 180 min was 23.2 µSv/h (SD=3.8, median=22, range=11-39).

The mean of maxEDR-1m at D1 was 9.2 µSv/h (SD=3.9, median=8, range=2-21).

### EDR-1m according to positioning the radiation detector

Of the 385 paired measurement, the pel-EDR-1m measurement was most frequently the greatest (maxEDR-1m); nevertheless in 105 paired measurements (27%) the st-EDR-1m was the greatest. In particular, considering the paired measurement performed at about 180 min, the maxEDR-1m was observed with the radiation detector facing the pelvis in 30 cases, facing the sternum in 12 cases, the two values being identical in 8 cases.

### Predictors of the variation of EDR-1m after administration of the radiopharmaceutical

The area under the ROC curve (AUC) was computed for all quantitative variables available prior to PRRT, as potential predictors in differentiating between inf-25@180 and sup-25@180 groups during the PRRT course.

The overall series was first studied: the greatest AUC was 0.89 for the creatinine clearance (p<0.001 vs. 0.5); just another AUC significantly greater than 0.5 was observed for KS (AUC=0.70, p<0.007 vs. 0.5) but significantly less than AUC of the creatinine clearance (p=0.007). A creatinine clearance value greater than 96 mL/min/1.7m^2^ prior to PRRT had a sensitivity (Se) of 27/32=84% (95% confidence interval CI: 67-95%), a specificity (Sp) of 15/18=83% (CI: 59-96%) and an accuracy (Acc) of 42/50=84% (CI: 71-93%), in predicting max-EDR-1m <25 µSv/h at 180 min after the end of the Lutathera infusion. The other quantitative variables, in particular age, body mass, BMI, GEP-NET grade and TMO were not significant predictors (Table 3).

By restricting the ROC analysis to the 24 initial PRRT cycles, the predictive value of creatinine clearance was even better, with an AUC of 0.98. A threshold greater than 91 mL/min/1.7m^2^ prior to PRRT, for predicting max-EDR-1m <25µSv/h at 180 min after the end of the Lutathera infusion, leaded to Se=15/17=88% (CI: 64-99%), Sp=7/7=100% (CI: 47-100%), Acc=22/24=92% (CI: 73-99%).

The KS had an AUC of 0.74 significantly greater than 0.5 but significantly less than that of creatinine clearance (p=0.01).

If the ROC analysis was restricted to the 30 PRRTs performed for midgut NETs, the best predictor was still creatinine clearance >95 mL/min/1.7m^2^ prior to PRRT, AUC=0.93, Se=18/21=86% (CI: 64-97%), Sp=8/9=89% (CI: 52-99%), Acc=26/30=87% (CI: 69-96%).

If the ROC analysis was restricted to the 19 PRRTs performed for foregut NETs, the best predictor was still creatinine clearance >95mL/min/1.7m^2^ prior to PRRT, AUC=0.83, Se=8/10=80% (CI: 44-97%), Sp=8/9=89% (CI: 52-99%), Acc=16/19=84% (CI: 60-97%).

### Comparison between the consecutive therapy courses in the patients who underwent several administrations of Lutathera

For the 26 PRRT administrations performed as 2^nd^ to 4^th^ cycles, three supplementary quantitative variables were considered: values observed during the previous PRRT cycle for the maxEDR-1m at 180 min and at D1 and the relative variation of the creatinine clearance between the two PRRT. There was no significant difference between the successive values in a given patient (Wilcoxon's test), for creatinine clearance (p= 0.61, mean and median variation=0% range -61% to +40%), for maxEDR-1m at 180 min (p=0.37) and at D1 (p=0.62).

Those three potential predictive variables were also added to the ROC analysis, but their AUC was less than that of the creatinine clearance determined before the current PRRT course.

### Predictive value on patient's outcome of the time course of ECR-1m after administration of Lutathera

During the follow-up period after their initial PRRT, 13 patients were alive after a mean of 857 days (median=873, range=188-1326), and 11 patients were deceased (6 with midgut NET and 5 another NET) after a mean period of 553 days (median 510, range=30-1328). The median overall survival for groups inf-25@180 and sup-25@180 was respectively 824 days (28 months) and 307 days (10 months) (Table 4).

**Figure 1:**
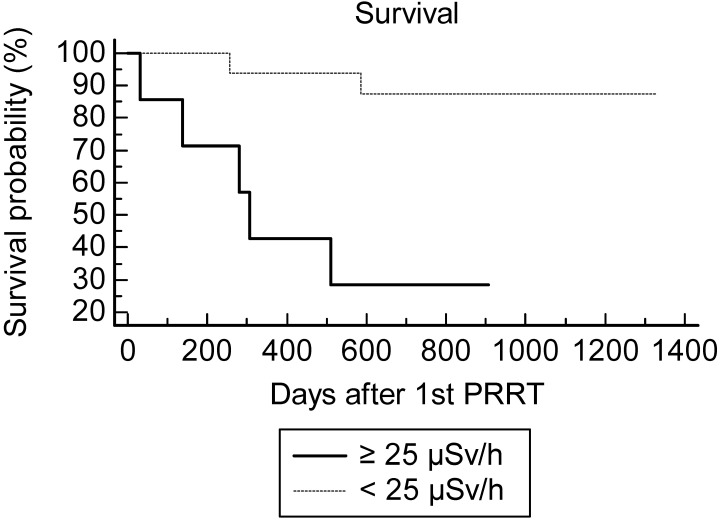
Survival after PRRT according to the EDR-1m 180 min after injection of 177Lu-DOTATATE during the 1st cycle.

Using the Kaplan-Meir analysis of the survival curve, there was a statistically significant difference of overall survival between the inf-25@180 (n=17) and sup-25@180 (n=7) groups at the 1^st^ PRRT cycles: hazard ratio=4.0 (CI 0.84-19), logrank test p=0.01 (Fig. 1).

One patient progressed after 2 PRRT cycles and was rapidly lost for follow-up. The rate of two-year survival after the 1^st^ PRRT was overall 16/23=70% (8/13 for midgut NET and 8/10 for other NETs). Concordantly with the Kaplan-Meir analysis of OS, the best predictor for two-year survival after the 1^st^ PRRT cycle was maxEDR-1m <25 µSv/h at 180 min (Table 4). Although the difference did not reach significance (p=0.2), the creatinine clearance appeared to be a less powerful predictor of survival, AUC=0.72 (vs 0.5 p=0.07). For none of the other quantitative variables the AUC was significantly greater than 0.5.

Four patients did not match the indication of the marketing authorization of Lutathera: non-GEP bronchial or ovarian NET in one patient each, and grade III pancreatic NET (panNET) in two patients. The patient with grade I bronchial NET had a favourable criterion at his first PRRT (maxEDR-1m <25µSv/h at 95 min) which became unfavourable at his 2^nd^ PRRT; alive after 2 years, she deceased 843 days after her 1^st^ PRRT cycle. The patient with a grade II ovarian NET had favourable criterion at her 1^st^ cycle and was alive 685 days after her single PRRT. One patient with a grade III panNET had a favourable criterion and was alive 824 days after his first PRRT, whereas the other patient with a grade III panNET had an unfavourable criterion and deceased 137 days after his single PRRT cycle.

The TFTV was determined on the ^68^Ga-DOTATOC PET/CT performed before the 1st PRRT cycle in the 8 patients from Hôpital St Antoine; mean = 214 cm^3^, median = 210, range = 80-350 (Table 5). As expected, there was a close correlation between TFTV and the KS (r= 0.85, p<0.01), but also the correlation between the TFTV and the max EDR-1m at 180min was close to statistical significance (r=0.62, p=0.1). In this subgroup, predicting the 2-year survival according to the max EDR-1m dose rate with the threshold of 25 µSv/h was correct in 7/8=88% patients (2 years after the 1st PRRT, 4 had survived and 4 deceased).

## Discussion

The primary objective of this study was to determine the durations of hospital stay required to obtain EDR-1m <40 µSv/h and <25 µSv/h, respectively, after administration of Lutathera in patients with a metastatic GEP-NET. We found that at 180 minutes after the end of the Lutathera infusion the EDR-1m was <40 μSv/h in all patients. This time point of 180 min was chosen because it corresponds in our practice to the end of the amino-acid infusion which lasts 4 h; the Lutathera infusion being started 30 min later and lasting 30 min, the amino-acid infusion is continued for 3 h after the end of the Lutathera administration and the patient cannot be discharged during that time frame.

The EDR-1m dropped <25 μSv/h at 180 min during 32 of 50 treatments (64%, CI: 49-77%).

In the study of Calais et al. 1, 76 patients were treated with a mean activity of 7580 MBq (SD = 338, range 6015-8395) of ^177^Lu-DOTATATE (average activity 2% greater than in our series). The mean time to observe EDR-1m <25 μSv/h in this series was 138 min vs. 141 min in the present study. According to Calais et al. 1 EDR-1m <25 μSv/h was obtained at 180 min in 54 patients (71%, CI: 60-81%), this proportion does not significantly differ from 64% in our series (Chi^2^=0.4, p=0.5). Calais et al. 1 continued EDR-1m measurements up to 6 h, achieving an EDR-1m <25 µSv/h in all patients, who were all discharged from the hospital without overnight stay. In our series, on D1 after an overnight stay, the mean EDR-1m was 9.2 µSv/h, the highest value being 21 µSv/h. The conclusion of Calais et al. was that ^177^Lu-DOTATATE radiopeptide therapy of NETs can be safely performed as an outpatient treatment on the majority of patients. Olmstead et al. observed in 10 patients a mean dose rate of 14 μSv/h at 4 h and 6.6 μSv/h at 20 h after therapy and concluded that, given the low dose rate and cumulative levels of radiation measured, the results support that an outpatient ^177^Lu-DOTATATE treatment protocol would not jeopardize public safety 19. Levart et al. 20 recently reported dose rate measurements during 13 Lutathera PRRTs in 11 patients who received a mean activity of 7700 MBq (range 7265-8038 MBq). The mean dose rate at 1 m from the anterior mid-abdomen was 15 μSv/h (range 5-25 μSv/h) when those patients were discharged at a mean time of 5.7 h (range 3.5-6.9 h) post-administration. The authors propose a dose constraint for relatives (family members) less than 5 mSv and less than 1 mSv per year for the public demonstrating that ^177^Lu-DOTATATE therapy can be administered safely on both an inpatient and outpatient basis. The independent results of the present study are concordant with the findings of those 3 studies and support their conclusions. If the EDR-1m of a patient after Lutathera administration falls relatively slowly, and provided that the facilities and logistics of the department are suitable, it may be possible to keep the patient as an outpatient in the department until the EDR-1m falls to the legal release limit.

In France as in many other countries, ^131^I therapy in patients with hyperthyroidism is performed on an outpatient basis. In 1993, O'Doherty et al. 21 reported an average EDR-1m of 0.006 µSv/h/MBq on the day of the administration of ^131^I for thyrotoxicosis, i.e. 36 µSv/h for 600 MBq. Recently, Liu et al. 22 concordantly found EDR-1m of 30 μSv/h, 4 h after administration of 340 MBq to 740 MBq of ^131^I. Considering radiation protection of the patient's carers, the recommendations for management of patients receiving Lutathera could follow those of ^131^I therapy of hyperthyroidism. The current suggestions about patient's subsequent behaviour to reduce risks from both external radiations and contamination to caregivers, his family and environment would become particularly important in particular in case of discharging earlier than one day after Lutathera administration. The patient will be encouraged to drink substantial quantities of water, in order to urinate as much as possible. He should limit contact with children and pregnant women and this interaction should be limited to less than 2 hours per day at a distance of less than 2 m. Pregnant partner or children should sleep in a separate bedroom from the patient for 2 days.

The first secondary objective aimed to evaluate whether there would be a difference in EDR-1m according to positioning the radiation detector facing the sternum or facing the pelvis of the patient. Although the drop in EDR-1m was usually slower in the pelvis leading to greater EDR-1m values, in 27% of measurements the EDR-1m value measured facing the sternum was the greatest, with a similar proportion in the late measurements at around 180 min. We therefore recommend to perform both measurements at each time point and to consider the highest of the paired values to maximize the risk evaluation. Calais et al. performed one single measurement facing the liver 1, which is likely to average the EDR-1m of the sternum and the pelvis but the present results favour a dual measurement to assess the maximal EDR-1m value in a given patient.

The second secondary objective was to find, among clinical, biochemical or imaging data collected before Lutathera PRRT in a given patient, predictors of the variation of EDR-1m after Lutathera administration. The aim is whether or not it is possible to predict when the patient's EDR-1m falls to below 25 µSv/h, and whether it is necessary to schedule for a given patient a hospital stay longer than 3 h after the end of Lutathera administration. We found that the best predictor for the possible discharging of a patient at 3 h, if this were to be authorized by the national authorities, was a value of creatinine clearance >96 mL/min/1.7m^2^ (positive predictive value PPV=27/30=90% CI: 73-98%). Thus, the capacity of renal excretion better predicted the pattern of the EDR-1m decrease than variables likely to reflect the retention of Lutathera, slowing down the drop of EDR-1m with time, such as the metastatic extent of the GEP-NET (TMO) or the uptake by the most intense target lesion (KS). It appeared that the accumulation of Lutathera in the target lesions and in non-target organs such as the spleen, the liver, the kidneys and the urinary bladder did not result in an EDR-1m> 40 µSv/h after 180 min in any patient, even with a low body mass or BMI. As a similar activity was administered to all patients, irrespective of the patients' BMI and body shape, it was expected that these may influence the EDR-1m to some degree, for example, there may be more attenuation of the EDR-1m in larger patients. However, no such relationship was found.

The third secondary objective was searching for variations between the consecutive PRRT cycles in the patients who underwent several administrations of Lutathera. However, no significant differences were found according to the criteria of external radiation exposure in a given patient. An interesting result of this survey is that the creatinine clearance, a strong predictor of the drop in EDR-1m, did not change significantly between the successive PRRT cycles, which may be seen as illustrating the efficacy of the renal protection in those patients with amino-acid infusion. A possible recruitment bias can be excluded, as no further retreatment with PRRT was excluded in any patient because of a decrease in renal function.

The final secondary objective was to check for a predictive value on patient's outcome of the decrease in the EDR-1m after administration of Lutathera, and we did find that this was the case. When the EDR-1m was <25 µSv/h at 180 min during the first PRRT cycle, two-year follow-up revealed that patient survival was likely, with a predictive value of 14/16 =86% (CI: 57-98%); 19 of the 23 patients whose two-year follow-up was available were correctly classified using this criterion. This criterion appeared also effective in the 4 patients who did not match the indication of the marketing authorisation of Lutathera (two non-GEP-NETs and two grade III GEP-NETs). It can be evaluated non-invasively by one single pair of external measurements at the first PRRT of metastatic GEP-NETs. It could be interesting to evaluate a similar criterion based on the measurements during the fourth PRRT cycle, which was not possible in this series since patients benefited from an uneven number of cycles during the inclusion time frame. The consistency of these findings should be assessed in a larger population in future studies. A potential mechanism to explain this predictive value could be the following: an EDR-1m less than 25 µSv/h at 180 min after the first administration means that the patient has cleared rather rapidly an important part of the injected activity. This may result from the interaction of two components: an effective renal function to clear the unbound radioactivity, which was the best determinant of EDR-1m < 25 µSv/h at 180 min and a limited tumor burden which retains radioactivity. In the limited group of 8 patients, this parameter showed a trend for correlation with TFTV, a more sophisticated evaluation of the NET extent than KS. It is likely that those two determinants predict a longer overall survival than deterioration of renal function and high NET burden.

### Limitations of the study

One limitation is the small number of patients in the cohort, due to the relatively rare occurrence of metastatic GEP-NETs.

Another limitation is the availability of pre-PRRT PET/CT imaging: ^68^Ga-DOTATOC PET/CT was only available for the 8 patients of Hôpital St Antoine, ^111^In-pentreotide SPECT/CT being available for the 16 patients of Institut Gustave Roussy. Thus, the intensity of labelled somatostatin analogue uptake by the target lesions could be evaluated in all patients only visually by means of the KS, instead of a more quantitative approach such as SUVmax or biological tumour volume. Furthermore, somatostatin analogue PET results in higher KS than derived from ^111^In-pentetreotide, particularly when lesions measure 2 cm or less 13, which may have induced some heterogeneity in the determination of the KS between the patients of the two centres. Nevertheless, KS was the only widely accepted score that can reflect the extent of the NET both on PET and SPECT, and the correlation of KS and TFTV, a quantitative approach in PET, was significant in only 8 patients, which is an argument favouring this option.

According to the study measurement protocol, the minimal number of paired measurements was 7 (every 30 min from 0 to 180 min post Lutathera infusion) which appeared to be insufficient for some patients, since the EDR-1m has fallen to <25 µSv/h after 180 minutes in only 32 of the 50 treatments. In many cases, it was necessary to make additional measurements after this time, but this criterion was still not reached in 11 therapy administrations during the measurement period. However, from the measurements reported by Calais et al. 1 over 6 h and the fact that EDR-1m was ≤21 µSv/h in all patients of our study on the next day, it can be anticipated that almost all patients could have been discharged from the hospital on the day of PRRT, based on their external radiation exposure.

## Conclusion

In this series of 50 PRRT therapy administration in 24 patients with midgut or foregut NETs, we observed that, 180 min after the administration of ca. 7400 MBq of Lutathera, EDR at 1 m from the patient was less than 40 µSv/h in all cases. A double determination of EDR at 1m facing the sternum and facing the pelvis should be preferred, since the value measured facing the sternum was the highest in a non-negligible number of cases. In case the regulations impose a maximal value for EDR-1m <25 µSv/h, our study found a reliable predictor at an individual level. The EDR-1m of patients whose creatinine clearance was greater than 96 mL/min/1.73m^2^ (or than 91 mL/min/1.73m^2^ for the first cycle of Lutathera) was most likely to drop below 25 µSv/h within 180 min, with a predictive value of 90% (CI: 73-98%). This means that the patients matching this criterion may be scheduled for discharge from the hospital 3 h after Lutathera administration, complying with a safe external radiation exposure.

Two interesting complementary results were also obtained. The creatinine clearance did not decrease significantly from one cycle of Lutathera to the next one, which is concordant with an absence of nephrotoxicity in this series and confirms the efficacy of the kidney protection by the amino-acids infusion. Finally, the patients whose EDR-1m dropped below 25 µSv/h at 180 min during their first PRRT were unlikely to decease during the two following years, with a predictive value of 88% (CI: 62-98%). The determination of this EDR-1m is a non-invasive and relatively simple practice, which furthermore is compulsory according to radioprotection regulations. As we observed, it may also yield prognostic information on overall survival. The observed difference of EDR-1m between short and long survivors might have been statistically significant just by chance but this observation deserves to be rechecked in a larger cohort.

## Figures and Tables

**Figure 2:**
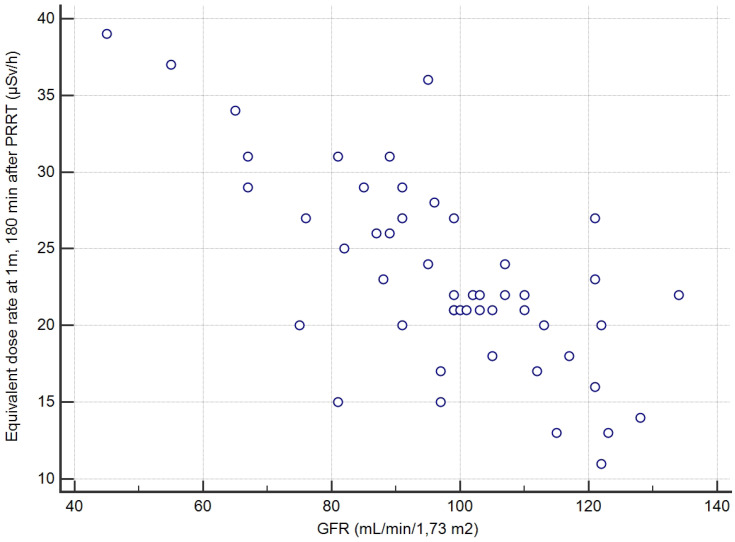
Correlation between the creatinine clearance and the EDR-1m at 180min based on the 50 cycles of measurement.

**Table 1:** Patients' characteristics

Characteristics	
Number of patients	24
**Gender**	
Male	10
Female	14
**Primitive NET**	
Midgut	13
Foregut	10
Other (ovary)	1
**Grade**	
I	11
II	11
III	2
**Krenning Score (KS)**	
1	2
2	7
3	10
4	5
**Metastatic organs**	
Liver	23
Lymph nodes	21
Peritoneum	20
Skeleton	17
Age (years) at initial PRRT: Mean, SD, median, (range)	59, 10.3, 59.5, (33-73)
Body mass (kg): Mean, SD, median, (range)	68.2, 14.3, 66, (47-103)
Body mass index (BMI) (kg/m^2^): Mean, SD, median, (range)	22.5, 2.4, 22.6, (18.9-27)
Creatinine clearance (mL/min/1.73m^2^): Mean, SD, median, (range)	97.7, 18.9, 99, (45-134)

**Table 2:** Main results of EDR-1m measurements (maxEDR-1m: greatest of the two measurements facing the sternum and facing the pelvis)

Characteristic	
Delay for maxEDR-1m <40 μSv/h (min): Number of cycles, mean, SD, median, (range)	n=50, 24.2, 37, 10, (0-180)
Delay for maxEDR-1m <25 μSv/h (min): Number of cycles, mean, SD, median, (range)	n=39, 141, 43, 146, (43-239)
maxEDR-1m at 180 min (μSv/h): Number of cycles with maxEDR-1m measured <25μSv/h following Lutathera administration, mean, SD, median, (range)	n=50, 23.2, 3.8, 22, (11-39)
maxEDR-1m after one overnight stay in hospital (D1) (μSv/h): Number of cycles, mean, SD, median, (range)	n=50, 9.2, 3.9, 8, (2-21)
Number of Lutathera PRRT cycles	50
Less than 25µSv/h at 180 min (inf 25@180)	32
Greater than or equal to 25µSv/h at 180 min (sup 25@180)	18

**Table 3:** Creatinine clearance as the best predictor among the quantitative variables for EDR-1m <25µSv/h at 180min after administration of the radiopharmaceutical

Parameter	Overall (N= 50 cycles)	Initial PRRT cycle (N= 24 patients)	Midgut NETs (N= 30 cycles)	Foregut NETs (N= 19 cycles)
AUC ROC	0.89	0.98	0.93	0.83
Cut-off value (mL/min/1.7m^2^)	>96	>91	>95	>95
Sensitivity [95% CI]	27/32=84% [67-95%]	15/17=88% [64-99%]	18/21=86% [64-97%]	8/10=80% [44-97%]
Specificity [95% CI]	15/18=83% [59-96%]	7/7=100% [47-100%]	8/9=89% [52-99%]	8/9=89% [52-99%]
Positive predictive value PPV [95% CI]	27/30=90% [73-97%]	15/15=100% [78-100%]	18/19=95% [74-100%]	8/9=89% [52-99%]
Negative predictive value NPV [95% CI]	15/20=75% [51-91%]	7/9=77% [40-97%]	8/11=73% [39-94%]	8/10=80% [44-97%]
Accuracy [95% CI]	42/50=84% [71-93%]	22/24=92% [73-99%]	26/30=87% [69-96%]	16/19=84% [60-97%]

AUC ROC: area under ROC curve, 95% CI: 95% confidence interval, NET: neuroendocrine tumour.

**Table 4:** Prediction of two-year survival according to EDR-1m during the initial PRRT

Parameter	n = 23 patients who either were alive 2 years after their initial PRRT or deceased from NET progression within 2 years
Two-year survival rate [95% CI]	16/23=65% [41-85%]
AUC ROC (p vs. AUC=0.5) Cut-off value	0.80 (p=0.004) EDR-1m <25µSv/h at 180 min during initial PRRT
Sensitivity [95% CI]	14/16=88% [62-98%]
Specificity [95% CI]	5/7=71% [29-96%]
Positive predictive value PPV (survival at 2 years) [95% CI]	14/16=88% [62-98%]
Negative predictive value NPV (decease within 2 years) [95% CI]	5/7=71% [29-96%]
Accuracy [95% CI]	19/23= 83% [61-95%]

AUC ROC: area under ROC curve, 95% CI: 95% confidence interval.

**Table 5:** NET SUVmax and total functional tumour volume of patients from hospital St Antoine. Relation with EDR-1m during the initial PRRT, Krenning score, and overall survival

Patient	NET grading	Metastatic sites	maxSUVmax	Total functional tumor volume (cm^3^)	KS	EDR-1m at 180 min (µSv/h)	Deceased	OS (days)
1	grade 2	Lymph nodes, liver, peritoneum, bone	17,7	130	2	11	no	821
2	grade 2	Lymph nodes, liver, peritoneum, bone	19	250	1	21	no	1246
3	grade 1	Lymph nodes, liver, bone	33	170	2	15	no	1326
4	grade 1	Lymph nodes, liver, peritoneum, bone	23	80	1	22	yes	1328
5	grade 1	Lymph nodes, liver, peritoneum, bone	23	170	2	17	yes	585
6	grade 2	Lymph nodes, liver, bone	8,8	270	4	39	yes	510
7	grade 1	Lymph nodes, liver, peritoneum, bone	33	290	4	37	yes	280
8	grade 3	Lymph nodes, liver, bone	32	350	3	27	yes	137

## Abbreviations

^68^Ga-DOTATOC: gallium-68 edotreotide
^177^Lu-DOTATATE: lutetium-177 oxodotreotide, granted a marketing authorisation as Lutathera
Acc: accuracy
ANSM: French national medicines agency
AUC: area under a ROC curve
BMI: body mass index
CI: 95% confidence interval
CT: X ray computed tomography
D1: the day following PRRT
EDR-1m: equivalent dose rate measured at 1 m of the patient
FDOPA: fluorine-18 dihydroxyphenylalanin
GEP‑NET: gastrointestinal neuroendocrine tumour
inf-25@180: group of PRRT cycles during which the max EDR-1m was less than 25 µSv/h, 180 min after ^177^Lu-DOTATATE administration
IRR17: European commission basic safety standards ionising radiations regulations 2017
KS: Krenning score
maxEDR-1m: the highest of the paired values, st-EDR-1m and pel-EDR-1m, at a time point in a patient
MRI: magnetic resonance imaging
NaI (Tl): crystal of sodium iodine including atoms of thallium
NET: neuroendocrine tumour
panNET: NET originating from the pancreas
PET: positron emission tomography
pel-EDR-1m: EDR-1m measured the detector facing the pelvis
PRRT: peptide receptor radionuclide therapy
ROC: receiver operating characteristic curve
SD: standard deviation
Se: sensitivity
Sp: specificity
sup-25@180: group of PRRT cycles during which the max EDR-1m remained greater than 25 µSv/h, 180 min after ^177^Lu-DOTATATE administration
st-EDR-1m: EDR-1m measured the detector facing the sternum
TMO: metastatic extent of the NET
